# The Impact of Interphase Precipitation on the Mechanical Behavior of Fire-Resistant Steels at an Elevated Temperature

**DOI:** 10.3390/ma13194294

**Published:** 2020-09-25

**Authors:** Jinghua Cong, Jiangwen Li, Jiajie Fan, Pengcheng Liu, Raja Devesh Kumar Misra, Chengjia Shang, Xuemin Wang

**Affiliations:** 1 Collaborative Innovation Center of Steel Technology, University of Science and Technology Beijing, Beijing 100083, China; congjinghua@126.com (J.C.); jiangwen0920@foxmail.com (J.L.); 17801055160@163.com (J.F.); pchliu@foxmail.com (P.L.); cjshang@ustb.edu.cn (C.S.); 2 Laboratory for Excellence in Advanced Steel Research, Department of Metallurgical, Materials and Biomedical Engineering, University of Texas at El Paso, 500 W. University Avenue, El Paso, TX 79968, USA; dmisra2@utep.edu

**Keywords:** thermo-mechanical controlled processing (TMCP), yield strength, interphase precipitation, precipitation strengthening, dislocation strengthening, fire-resistance

## Abstract

In this study, we address the challenge of obtaining high strength at ambient and elevated temperatures in fire-resistant Ti–Mo–V steel with ferrite microstructures through thermo-mechanical controlled processing (TMCP). Thermally stable interphase precipitation of (Ti, Mo, V)C was an important criterion for retaining strength at elevated temperatures. Electron microscopy indicated that interphase precipitation occurred during continuous cooling after controlled rolling, where the volume fraction of interphase precipitation was controlled by the laminar cooling temperature. The interphase precipitation of MC carbides with an NaCl-type crystal structure indicated a Baker–Nutting (B–N) orientation relationship with ferrite. When the steel was isothermally held at 600 °C for up to 3 h, interphase precipitation occurred during TMCP with high thermal stability. At the same time, some random precipitation took place during isothermal holding. The interphase precipitation increased the elastic modulus of the experimental steels at an elevated temperature. It is proposed that fire-resistant steel with thermally stable interphase precipitation is preferred, which enhances precipitation strengthening and dislocation strengthening at elevated temperatures.

## 1. Introduction

Precipitation strengthening is an effective approach to increase the strength of steels and has received significant attention. Since its discovery in the 1960s [1], interphase precipitation has been an important form of precipitation and of particular interest to researchers [2,3,4]. Studies have shown that a number of precipitate-forming elements can form interphase precipitation, such as carbonitrides of Ti, Nb, V, Mo, Cr, etc. [5,6,7,8,9,10,11,12]. There have been two main approaches to obtain interphase precipitation up to now. One is isothermal treatment and the other is continuous cooling [5,6,7,13,14,15]. In addition, some studies have shown that interphase precipitation always takes place at the beginning of phase transformation during isothermal treatment [16]. A study has also shown that interphase precipitation is easier at higher temperatures during the continuous cooling process [17]. In fact, a reasonable match between the migration rate of the interface and the enrichment of precipitating elements can effectively promote the nucleation of interphase precipitation. Moreover, there are many studies on the orientation relationship between interphase precipitation and ferrite matrix, generally showing a Baker–Nutting (B–N) orientation relationship at the beginning of interphase precipitation. However, with the coarsening of previous interphase precipitation, it gradually changes to a Nishiyama–Wassermann (N-W) orientation relationship [18]. In terms of mechanical properties, the influence of interphase precipitation on formability and resistance to hydrogen embrittlement was also reported [19,20], but the majority of the studies on interphase precipitation mainly focused on its contribution to strength at room temperature. It is worth stressing that there are no relevant studies on the influence of the stability of precipitates on mechanical properties at elevated temperatures.

The fire-resistance of steel is generally measured at 600 °C after being held at 600 °C for 3 h [21,22,23,24,25,26]. Generally, the ratio of yield strength at 600 °C to that at room temperature is used to evaluate the steel’s fire-resistance. When steels are used at elevated temperatures, reduction in strength is caused by many factors, such as the reduction in Peierls-Nabarro stress, dislocation climb/cross-slip, precipitate coarsening, etc. [21]. It is widely recognized that precipitation strengthening is an important approach to improve the strength of steel at elevated temperatures. The supersaturated precipitates in ferrite can effectively improve the fire-resistance of steel by precipitation during reheating [22,23,24,25,26]. However, it is difficult to simultaneously improve the strength of steel at room temperature and at elevated temperatures. Some studies have shown that a large amount of interphase precipitation occurs during air cooling after rolling [13] and its effect on strengthening contribution is up to ~300 MPa [5]. However, obtaining high strength at ambient and high temperatures is a challenge.

The objective of the present study is to explore the effect of interphase precipitation on the fire-resistance of steel. By studying high temperature thermal stability of interphase precipitation and the relationship between the morphology, distribution and size of interphase precipitation and thermo-mechanical controlled processing, the contribution of interphase precipitation to the strength of materials at elevated temperatures is revealed. The findings from the present study may provide an alternative approach for improving the fire-resistance of steel.

## 2. Materials and Methods

Table 1 shows the chemical composition of the experimental steel.

**Figure 1:**
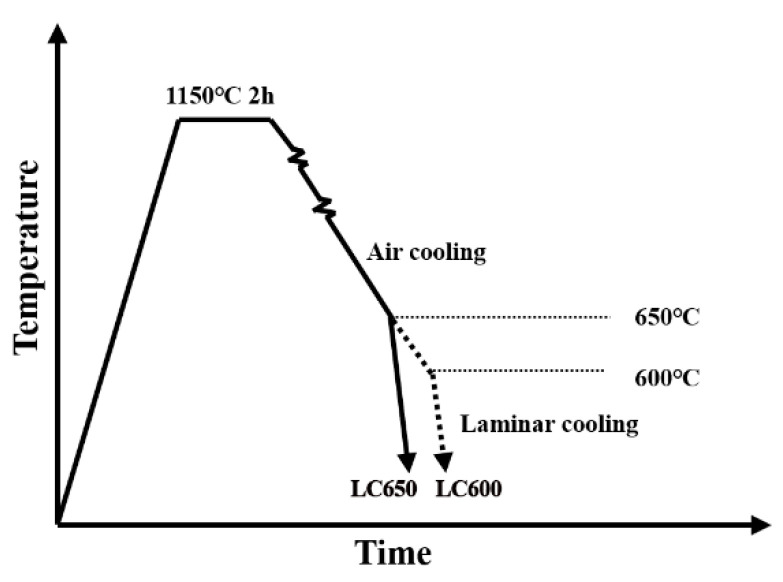
Thermo-mechanical controlled processing (TMCP) process flow chart of experimental steels.

Steel was made in a 10 kg vacuum induction furnace, followed by forging and cutting it into a rectangular ingot with 100 mm thickness, 100 mm width and 100 mm length. The steel was soaked at 1150 °C for 2 h to dissolve microalloying elements and subsequently rolled to 12 mm via multiple passes between 1100 °C and 820 °C, from the austenite recrystallization region to the austenite non-recrystallization region. After rolling, the steel plates were cooled to 650 °C and 600 °C, respectively, in air (1 °C/s ≤ the cooling rate ≤ 2 °C/s), followed by laminar cooling (the cooling rate ≥10 °C/s) to ambient temperature, as shown in Figure 1. The two specimens are referred to hereafter as LC650 and LC600 (laminar cooling), respectively. At the same time, in order to study the morphology, distribution and size of precipitates in fire-resistance testing, the specimens of steel LC650 and steel LC600 were held at 600 °C for 3 h. The tempered samples are hereafter referred to as LC650+600T and LC600+600T.

Specimens for microstructural studies were polished using a standard metallographic procedure and etched with 3% nital solution and observed using an optical microscope (OM) (OLYMPUS, Tokyo, Japan). Microhardness measurements of ferrite were taken randomly using a Vickers hardness tester (CAS, Shanghai, China) with a load of 0.1 kg. Transmission electron microscopy (TEM) (JEOL, Tokyo, Japan) studies were conducted using 3 mm diameter thin foils and carbon extraction replicas. The former were mechanically thinned to 0.05 mm and electropolished using a solution of 5% perchloric acid and 95% alcohol at −25 °C. The latter were prepared by light etching with 2% nital solution to determine the size and volume fraction of precipitates. The extraction replica samples and thin foil samples were examined by JEOL JEM-2100 TEM (JEOL, Tokyo, Japan)) at an acceleration voltage of 200 kV. Digital Micrograph software (GATAN, Pleasanton, CA, United States) was used to accomplish fast Fourier transform (FFT) and inverse fast Fourier transform (IFFT). The X-ray tube was operated at 40 kV and 40 mA. The X-ray diffraction data were recorded from 2θ = 40° to 100° with a step of 0.02° to obtain diffraction peaks of (110), (200), (211) and (220).

Specimen blanks for tensile specimens were cut from the rolled plates in the transverse direction. The tensile tests were conducted at room temperature and at 600 °C, according to the Chinese standards GB/T 228-2002 and GB/T 4338-2015, respectively [27,28]. Two samples were tested for each experimental steel at each testing temperature and the average values were taken for the results of tensile tests. Round bar tensile specimens with a 5 mm gage diameter and 25 mm gage length were tensile tested at room temperature at a crosshead speed of 1 mm/min. Samples of identical dimensions were tested at an elevated temperature. They were kept at 600 °C for 3 h prior to the elevated temperature tensile test at 600 °C at a crosshead speed of 0.5 mm/min.

## 3. Results

**Figure 2:**
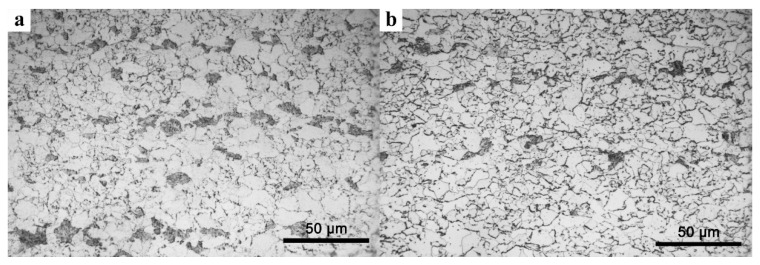
Optical micrographs of as-rolled steel with a start laminar cooling temperature of 650 °C (**a**) and 600 °C (**b**).

Optical micrographs of specimens in the as-rolled state, whose start laminar cooling temperatures were 650 °C and 600 °C, respectively, are presented in Figure 2. The microstructure was characterized by ferrite with a small amount of bainite. In Figure 2, the gray microstructure is ferrite and the black microstructure is bainite. When the start laminar cooling temperature was decreased, the volume fraction of ferrite was increased. The volume fractions of ferrite were 91% (steel LC650) and 97% (steel LC600). There was little difference in ferrite grain size between steel LC650 and steel LC600, which were ~5.91 μm and ~5.85 μm, respectively. In addition, bainite transformed by laminar cooling of untransformed austenite from 650 °C and 600 °C, respectively.

**Figure 3:**
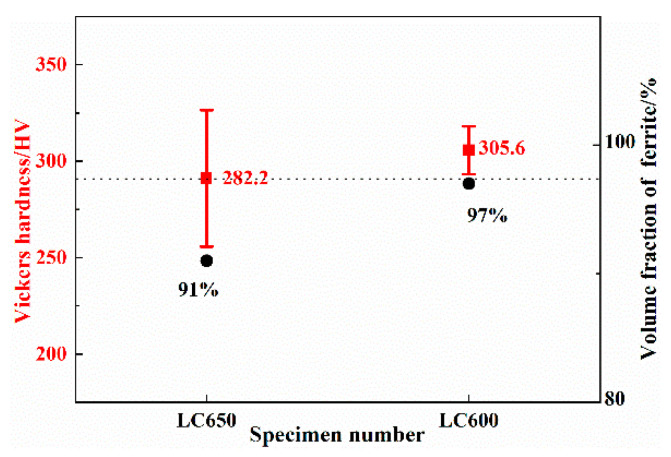
Vickers hardness and ferrite volume fraction in experimental steels.

The change in Vickers hardness of ferrite obtained by different TMCP processing is presented in Figure 3. The upper and lower limits of the error bar represent the maximum and minimum values of the measurements with 95% confidence interval, respectively. It can be seen from the figure that the average hardness of ferrite increased with the decrease in start laminar cooling temperature and the fluctuation of hardness data was minimal for sample LC600. The increase in the hardness of ferrite is discussed later.

**Figure 4:**
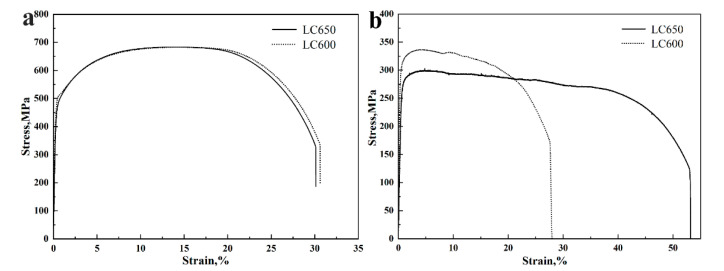
Tensile stress–strain curve of experimental steels at room temperature and at 600 °C. (**a**) Tensile test at room temperature, and (**b**) tensile test at 600 °C.

Table 2 summarizes the mechanical properties of the experimental steels at room temperature and an elevated temperature. It can be seen from the table that with the decrease in start laminar cooling temperature, the yield strength at room temperature was increased. A similar trend was observed for yield ratio and yield strength at the elevated temperature. Both of them have excellent low temperature toughness, and their impact energy at −40 °C is greater than 200 J. It is evident that the yield strength of steel LC600 was superior to that of steel LC650 at an elevated temperature. The stress–strain curves at room temperature and an elevated temperature are presented in Figure 4. It may be noted that with the increase in the volume fraction of ferrite, the elongation gradually increased with the decrease in start laminar cooling temperature. The low-temperature toughness of the experimental steels was excellent because of the large amount of ferrite constituents.

## 4. Discussion

From the OM micrographs (Figure 2), it can be seen that ferrite and bainite were the main constituents. According to the transformation law, during the continuous cooling process, the volume fraction of ferrite can be tuned by controlling the start laminar cooling temperature. The untransformed austenite transformed into bainite during the laminar cooling stage after air cooling. The mechanical properties at room temperature and elevated temperature are shown in Table 2. From Table 2 and Figure 2, it can be seen that with the decrease in laminar cooling temperature, the volume fraction of bainite was decreased and the tensile strength of the experimental steel at room temperature was decreased, while the yield strength at room temperature was increased. The difference between yield strength at room temperature and yield strength at the elevated temperature was smaller when the start laminar cooling temperature was 600 °C. It can also be seen from the stress–strain curve in Figure 4 that the yield strength at room temperature has little difference. However, the yield strength at the elevated temperature, with a lower laminar cooling temperature, is significantly higher. We predict that interphase precipitation is the main reason for superior fire-resistance, which will be confirmed below.

From Figure 3, it can be seen that the hardness of ferrite increased gradually with the decrease in start laminar cooling temperature. It is known that hardness of ferrite depends on its carbon-content, density of dislocations and precipitation. In our study, the carbon content in specimens is expected to be similar. Thus, the difference in hardness is determined by the density of the precipitated carbides and dislocations in polygonal ferrite.

**Figure 5:**
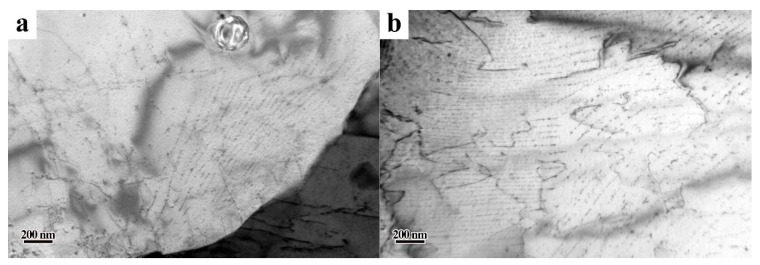
TEM micrographs showing carbides in as-rolled steel. (**a**) Steel LC650 and (**b**) steel LC600.

**Figure 6:**
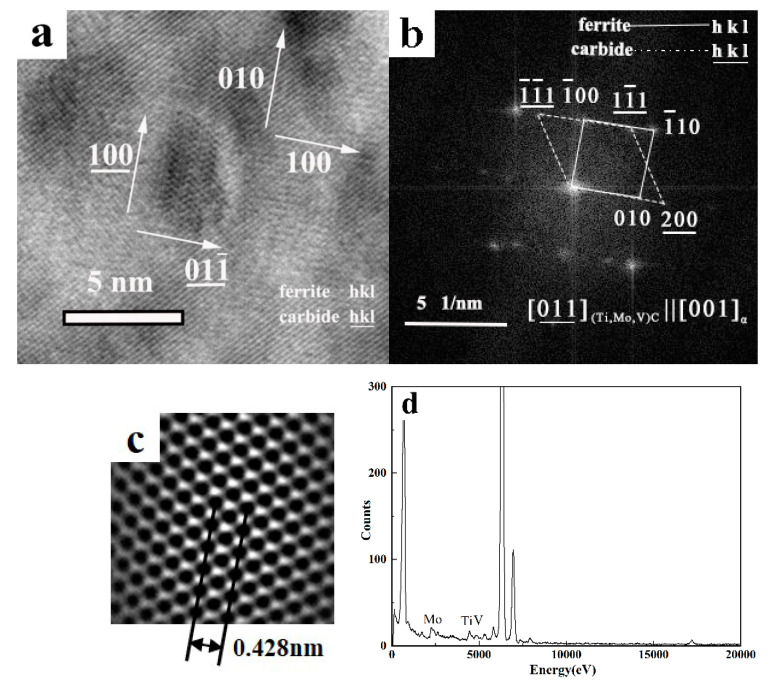
HRTEM images of nanometer-sized carbides in steel LC600. (**a**) High resolution image of interphase precipitation, (**b**) corresponding fast Fourier transformation (FFT) diffractogram, (**c**) inverse fast Fourier transformation (IFFT) lattice image of interphase precipitation, and (**d**) EDS (Energy-dispersive X-ray spectroscopy) analysis of interphase precipitation.

As shown in Figure 5, there was a high degree of interphase precipitation in the hot-rolled microstructure. It can be clearly seen that interphase precipitation in steel LC600 was mainly present in ferrite grains. In addition to some interphase precipitate regions, there were also some precipitate-free regions in the ferrite grains of steel LC650. This is the reason why the range of Vickers hardness fluctuation in the ferrite in steel LC650 in Figure 3 was large, whereas that of the ferrite in steel LC600 was small. From the observations in Figure 5 and Figure 6, the size of interphase precipitates was less than ~10 nm and the precipitates were mainly composed of Mo, Ti and V. It can be seen from Figure 5 that with the decrease in start laminar cooling temperature, the spacing between the rows of interphase precipitation was gradually decreased. The row spacing of interphase precipitation (i.e., the minimum ledge height) is proportional to the interfacial energy of the interface and inversely proportional to the chemical free energy change per unit volume (i.e., the driving force) [29,30]. Thus, the row spacing of interphase precipitation decreases because of lower phase transformation temperature and consequent increase in driving force.

**Figure 7:**
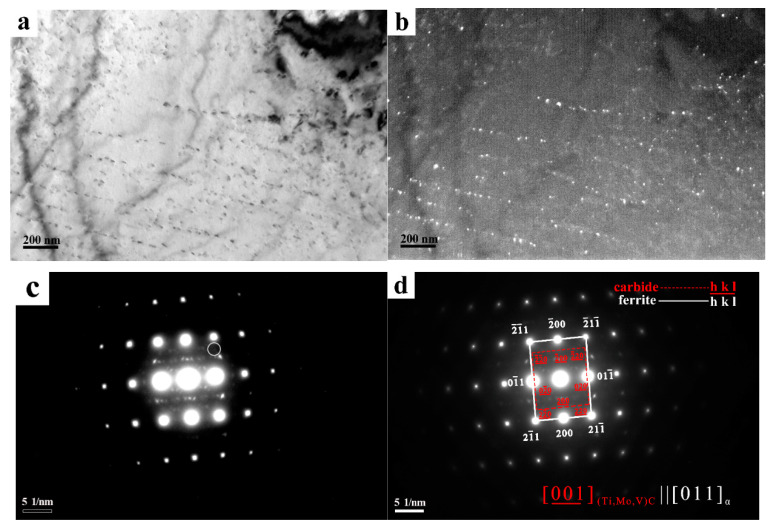
TEM micrographs of steel LC600. (**a**) Bright-field image, (**b**) dark-field image, (**c**) the selected area diffraction pattern (SADP) of interphase precipitation and matrix, (the red circle in figure is the spot selected by the dark-field image) and (**d**) the carbides adapt only one variant of the Baker–Nutting orientation relationship.

Given that the interphase precipitation occurs at the interface of γ→α transformation, it is nearly parallel to the advancing γ→α transformation front. The selected area diffraction patterns (SADP) and the corresponding analysis in Figure 7 revealed that MC carbides with an NaCl-type crystal structure had the following orientation relationship with ferrite:

(1 0 0) _MC_// (1 0 0) _Ferrite_

[0 0 1] _MC_// [0 1 1] _Ferrite_

The carbides obeyed the Baker–Nutting (B–N) orientation relationship with respect to the ferrite matrix. An example of high-resolution TEM (HRTEM) study of nanometer-sized carbides is presented in Figure 6. The corresponding diffraction pattern obtained using a 2-dimensional fast Fourier Transformation of the image revealed that the carbides exhibited a B–N orientation relationship ((1 0 0) _MC_// (0 1 0) _Ferrite_, [0 1 1] _MC_// [0 0 1] _Ferrite_) with respect to the ferrite matrix. Therefore, the interphase precipitation had a greater consistency with ferrite in the formation of the ferrite/austenite interface [6]. Moreover, by using an inverse fast Fourier Transformation (IFFT) for carbides, from the lattice image of carbide (presented in the inset in Figure 6), the lattice parameter was determined to be 0.428 nm and was less than the lattice constant of TiC. This is the reason for the constituent elements of precipitates to predominantly contain Ti, though they also contained V and Mo, as shown in Figure 6. The atomic radius of Mo or V is smaller than Ti, so the partial replacement of Ti renders the lattice parameter of precipitates to be smaller.

**Figure 8:**
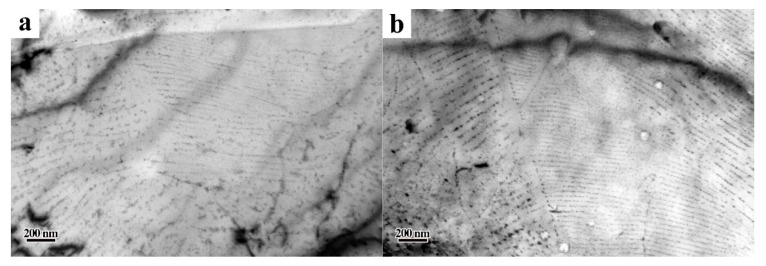
TEM micrographs showing carbides in steel isothermally treated at 600 °C for 3 h. (**a**) Steel LC650+600T and (**b**) Steel LC600+600T.

As shown in Figure 5, there were a large number of precipitates. From Figure 8, it can be seen that after being held at an elevated temperature for 3 h, the size of the interphase precipitates was still smaller than ~20 nm, with no apparent growth or coarsening. Given that the precipitates are obstacles to the movement of dislocations, the experimental steels showed higher strength at elevated temperature. For interphase precipitation, which mainly comprised of Ti, Mo and V, there was no obvious growth or coarsening when held at 600 °C for 3 h. As mentioned in reference [31], this will reduce the lattice constant of TiC and render it coherent with the matrix, when Mo replaces Ti in TiC. The partial replacement of Ti by Mo during the formation of TiC particles can decrease the strain energy and keep the interface coherent with B–N orientation relationship. Thus, the coarsening of interphase precipitation in the experiment steels was difficult when held at 600 °C for 3 h.

It can be seen from the TEM observation that the interphase precipitation has superior thermal stability at an elevated temperature. Consequently, the strength contribution of interphase precipitation at elevated temperatures will be obvious. To further verify this standpoint, we will estimate each strength contribution later.

In order to calculate the strength contribution of precipitates, volume fraction and average diameter need to be determined. The volume fraction $f_{V}$ of precipitates was estimated from the following Equation (1) [32]. The average diameter *d* of precipitates was measured by Image-Pro Plus software with TEM micrographs. The results are shown in Table 3.

(1) $$f_{V}=\frac{\frac{4}{3}\pi {\left(\frac{d}{2}\right)}^{3}\cdot n}{V}=\frac{2}{3}d\cdot \frac{f_{S}}{h}$$

where $f_{S}$ is the area percentage of precipitates in the TEM image, *d* is the average diameter of precipitates, n is the number of the precipitates in the TEM image, *V* is the volume fraction of the TEM carbon extraction replicas specimens and *h* is the thickness of carbon extraction replicas (the value here is 50 nm)

The strength at room temperature is the sum of different strengthening mechanisms, which can be obtained from reference [33]. These include friction stress of the ferritic matrix, solid-solute strengthening and fine-grain strengthening (Equation (2)). It is generally known that good mechanical properties depend on the reasonable balance between intragranular strength and grain-boundary strength. Since grain boundary strength is higher than intragranular strength at room temperature, the strengthening effect of grain refinement is obvious. However, with the increase in temperature, grain-boundary strength decreases gradually and intragranular strength increases. Therefore, the grain boundary strength will be equal to or even lower than the intragranular strength at an elevated temperature. It was also confirmed by Sha et al. [34] that ferritic grain-boundary would start to slip. Therefore, grain refinement has an insignificant strengthening effect on the strength of ferritic steel at an elevated temperature. The fine-grained strengthening contribution to $\Delta \sigma_{GB}$ in Equation (2) can be neglected and Equation (3) is obtained.

(2) $$\Delta \sigma_{y}=\Delta \sigma_{0}+\Delta \sigma_{SS}+\Delta \sigma_{GB}\;=\;15.4[3.5+2.1\left(\mathrm{Mn}\right)+5.4\left(\mathrm{Si}\right)+23.4\left(C\right)+23(N_{f})+1.13D^{-1/2}]$$

(3) $$\Delta \sigma_{y}=\Delta \sigma_{0}+\Delta \sigma_{SS}=15.4[3.5+2.1(\mathrm{Mn})+5.4(\mathrm{Si})+23.4(C)+23(N_{f})]$$

where $\Delta \sigma_{0}$ is the friction of the ferritic matrix, $\Delta \sigma_{SS}$ is the solid-solute strengthening and $\Delta \sigma_{y}$ is the sum of $\Delta \sigma_{0}$, $\Delta \sigma_{SS}$ and $\Delta \sigma_{GB}$. Since the experimental steels had identical compositions with different controlled cooling processes, there was little difference in the solid-solute strengthening and friction stress of the ferritic matrix. So, $\Delta \sigma_{y}$ is disregarded in this study when discussing the contribution of strength at elevated temperature. The total yield stress $\sigma_{y}$ can be estimated by Equation (4). The strength contribution $\Delta \sigma_{Dis+Orowan}$ is the sum of dislocation strengthening and precipitation strengthening [35], as shown in Equation (5). The strengthening contribution of dislocation strengthening is shown in Equation (6) [7].

(4) $$\sigma_{y}=\Delta \sigma_{y}+\Delta \sigma_{Dis+Orowan}$$

(5) $$\Delta \sigma_{Dis+Orowan}=\sqrt{\Delta \sigma_{Dis}^{2}+\Delta \sigma_{Orowan}^{2}}$$

where $\Delta \sigma_{Dis}$ is the dislocation strengthening and $\Delta \sigma_{Orowan}$ is the precipitation strengthening.

The elastic modulus *E* is calculated from the stress–strain curves at room temperature and elevated temperature. Given that it does not change appreciably with the increase in temperature, the Poisson’s ratio υ is 0.291 [36]. The shear modulus *G* at room temperature and elevated temperature can be calculated by *E* and υ. The results are shown in Table 4. The elastic modulus of steel LC600 (177.2 GPa) at room temperature is similar to that of steel LC650 (177.0 GPa), but its elastic modulus (136.5 GPa) at an elevated temperature is obviously higher than that (105.6 GPa) of the latter. The considerable difference between steel LC600 and steel LC650 is their volume fraction and row spacing of interphase precipitation. This means that interphase precipitation has a significant effect on the elastic modulus at an elevated temperature.

(6) $$\Delta \sigma_{Dis}=\alpha MGb\sqrt{\rho }$$

where α is constant with value of 0.435, M is the Taylor factor and for ferritic steel is 2.75, *G* is the shear modulus (Table 4), b is the Burgers vector and is 0.248 nm and *ρ* is dislocation density (Table 3). The calculation of dislocation density is based on XRD results and Equation (7) [37]. The results are shown in the last column of Table 3.

(7) $$\rho \;=2\sqrt{3}\varepsilon /Db$$

where *ρ* is dislocation density, *ε* is X-ray wave strain, b is the Burgers vector and is equal to 0.248 nm and *D* is the average particle size.

**Figure 9:**
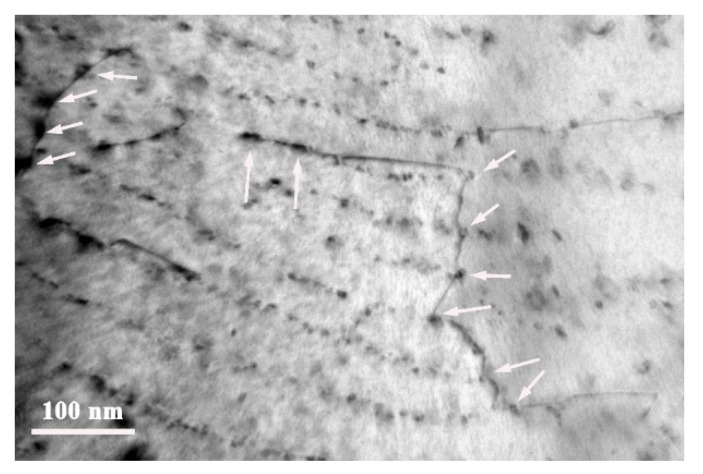
TEM micrographs of dislocations and precipitates in steel LC600+600T.

It can be seen from Table 3 that the dislocation density of steel LC600 (5.07 × 10^13^ m^−2^) was lower than that of steel LC650 (9.99 × 10^13^ m^−2^). This is mainly due to the difference in bainite content between the two steels. After being held at 600 °C for 3 h, the dislocation density of steel LC650+600T decreased to 6.00 × 10^13^ m^−2^, which was a 39.9% decrease, but that of steel LC600+600T only decreased to 3.92 × 10^13^ m^−2^, which was a 22.7% decrease. This is not difficult to understand, mainly because there was a higher volume fraction of interphase precipitates in steel LC600 than in steel LC650. It can also be recognized from Figure 9 that interphase precipitation can effectively prevent annihilation of the dislocations at 600 °C.

To estimate the contribution of precipitation strengthening, both interphase precipitation and random precipitation need to be considered. A number of studies [7,38] showed the contribution of interphase precipitation to strengthening. However, because of large amount of interphase precipitation and random precipitation in the experimental steels, the contribution of overall precipitation strength cannot be calculated separately. Therefore, the Ashby–Orowan relationship was used to calculate the contribution of precipitation to strengthening [39], given by Equation (8):

(8) $$\Delta \sigma_{Orowan}=\;0.3728\frac{Gb}{K}\times \frac{f^{\frac{1}{2}}}{d}\ln(\frac{1.2d}{2b})$$

where K is a constant, *d* is the average diameter of precipitates, f is the area percentage of the precipitates (equivalent to $f_{V}$ in Table 3), b is the Burgers vector and is 0.248 nm and *G* is the shear modulus, shown in Table 4.

The contribution of strengthening at room temperature in Table 5 shows that there is little difference between $\Delta \sigma_{Dis+Orowan}$ (steel LC600) and $\sigma_{Dis+Orowan}$ (steel LC650). However, due to the higher volume fraction of bainite, the measured dislocation density of steel LC650 will be higher than the actual dislocation density. Therefore, it is not difficult to understand why the yield strength of steel LC650 is lower than that of steel LC650 at room temperature. $\Delta \sigma_{Dis+Orowan}$ (steel LC600+600T) ~165.4 MPa is significantly higher than $\Delta \sigma_{Dis+Orowan}$ (steel LC650+600T) ~137.8 MPa. This is the reason why the properties of steel LC600 with more interphase precipitation at an elevated temperature were superior to steel LC650.

**Figure 10:**
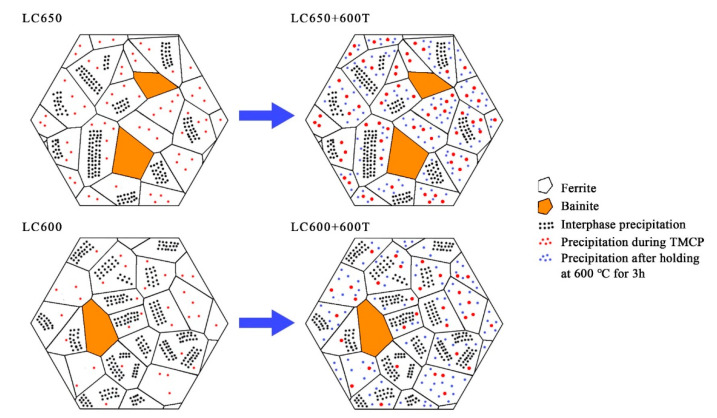
Schematic illustration of changes in the morphology and nature of precipitates during different processes.

In conclusion, the fire-resistance of experimental steels is closely related to the amount of interphase precipitation. The changes in the morphology and nature of precipitates before and after tempering are shown in Figure 10. Based on the strength contribution calculated above, it can be seen that the volume fraction of precipitates in the experimental steels after tempering was not so different. The experimental steel with more interphase precipitation had superior mechanical properties at elevated temperature.

## 5. Conclusions

Although the start laminar cooling temperatures were different, the yield strengths of the steels were greater than 460 MPa and the elongations were greater than 26%. The impact energy at −40 °C was greater than 200 J. The lower the start laminar cooling temperature, the more interphase precipitation occurred and the higher the strength of ferrite was. After being held at 600 °C for 3 h, the interphase precipitates continued to be fine, and this effectively prevented annihilation of the dislocations and greatly improved the fire-resistance of steels.

The interphase precipitation of experimental steels followed B-N orientation relationship, with NaCl structure. The Ti precipitates had some Mo and V.

The calculation indicated that precipitation strengthening and dislocation strengthening contributions of steels LC650 and LC600 at elevated temperatures were ~137.8 MPa and ~165.4 MPa, respectively. When the start laminar cooling temperature was decreased, the contribution of precipitation strengthening was increased. The high fire-resistance of the steels was mainly because of interphase precipitation that did not coarsen and the pinning effect of interphase precipitates prevented annihilation of the dislocations at elevated temperature.

## Figures and Tables

**Table 1:** The chemical composition of the experimental steel (wt.%).

C	Mn	Si	Cr	Mo	Nb	Ti	V	Cu	Ni	P	S
0.06	1.30	0.18	0.40	0.24	0.03	0.13	0.10	0.56	0.32	0.012	0.008

**Table 2:** Mechanical properties of the experimental steels.

Temperature	Room Temperature				−40 °C	Tensile of 600 °C	
Mechanical Property	YS (MPa)	TS (MPa)	Yield Ratio	El (%)	CVN (J)	YS (MPa)	TS (MPa)
Steel LC650	476.8	686.2	0.69	30.9	280	228.4	303.1
Steel LC600	494.9	677.7	0.73	31.4	252	288.4	337.3

(YS: yield strength; TS: tensile strength; El: elongation; CVN: Charpy v-notch impact toughness).

**Table 3:** The density of precipitates and dislocations at room temperature and elevated temperature.

Process	*d*/nm	$f_{S}\;$	$f_{V}\;$	*ρ*/m^−2^
Steel LC650	5.70 ± 0.73	0.0204	0.0016	9.99 × 10^13^
Steel LC600	6.45 ± 0.69	0.0367	0.0032	5.07 × 10^13^
Steel LC650+600T	6.53 ± 0.41	0.0314	0.0027	6.00 × 10^13^
Steel LC600+600T	5.95 ± 0.41	0.0320	0.0025	3.92 × 10^13^

**Table 4:** The elastic modulus and shear modulus at room temperature and an elevated temperature.

Process	*E* (600 °C)/GPa	*E* (RT)/GPa	υ	*G* (600 °C)/GPa	*G* (RT)/GPa
Steel LC650	105.6	177.0	0.291	40.9	68.6
Steel LC600	136.5	177.2	0.291	52.9	68.6

**Table 5:** Precipitation strengthening and dislocation strengthening contributions at room temperature and elevated temperature.

Process	$\Delta \sigma_{\mathrm{Dis}}/\mathrm{MPa}$	$\Delta \sigma_{\mathrm{Orowan}}/\mathrm{MPa}$	$\Delta \sigma_{\mathrm{Dis}+\mathrm{Orowan}}/\mathrm{MPa}$
Steel LC650	203.2	138.7	246.0
Steel LC600	145.0	183.2	233.6
Steel LC650+600T	94.0	100.8	137.8
Steel LC600+600T	98.2	133.1	165.4
